# Data on Vietnamese patients׳ behavior in using information sources, perceived data sufficiency and (non)optimal choice of health care provider

**DOI:** 10.1016/j.dib.2016.04.066

**Published:** 2016-05-10

**Authors:** Quan Hoang Vuong

**Affiliations:** Centre Emile Bernheim, Solvay Brussels School of Economics and Management, Université Libre de Bruxelles, 50 Ave. F.D. Roosevelt, Brussels B-1050, Belgium

## Abstract

This data article introduces a data set containing 1459 observations that can enable researchers to examine issues related to and perform statistical investigations into questions of relationships between sources of health care information, data sufficiency, trust levels between patients and healthcare experts (and the advice). The data set also records assessment of Vietnamese patients on whether their choice of health care provider is best available (optimal vs. nonoptimal). The data come from a survey in many hospitals in Hanoi and several neighboring provinces/cities in the North of Vietnam, during the last quarter of 2015. Variables that can be useful for future analysis include sources and availability of information, cost, and amount of time for seeking information. The quality of information and health professionals’ credibility are critical factors in helping patients choose a health care provider.

Mendeley Data, v1 http://dx.doi.org/10.17632/gmbz53tpwc.1; and can enable the modeling after useful discrete data models such as BCL, with one example being provided in this data article.

**Specifications Table**

**Table:** 

Subject area	*Medical*
More specific subject area	*Health care information, patients’ assessment of data sufficiency and (non)optimal behavior and choice in choosing health care providers for their medical needs*
Type of data	*Table, text file, graph*
How data was acquired	*Survey*
Data format	*Raw, filtered, and partially analyzed*
Experimental factors	*Raw data obtained from a survey patients at hospitals and clinics in Hanoi and several neighboring provinces, in the North of Vietnam*
Experimental features	*The experiment focuses on observations information demand, data sufficiency and efficiency in Vietnamese patients׳ choice of health care provider*
Data source location	*Bach Mai, Viet Duc, Thanh Nhan Hospitals, Hanoi, Vietnam (and others, see*Appendix A)
Data accessibility	*Datasets are provided with this article*.

**Value of the data**

• The data help acquire understanding about patients’ demand for health information before choosing health care provider.
• Assessments of patients access to different sources of information and data, and values in their decision making process.
• The data enable researchers’ further examination into alternative functions of available but seemly underutilized public information system and health service such as the public emergency medical service hot line 115.
• The data potentially offer an opportunity of examining the quality of medical information from different sources and perception of efficiency in Vietnamese patients’ choice of health care provider.

## 1 Data

The data set contains 1459 records obtained from a survey of assessments from Vietnamese patients about information sources, time consumption and labor cost for acquiring information, the perceived value of information and efficiency in choice of health care provider.

**Fig. 1:**
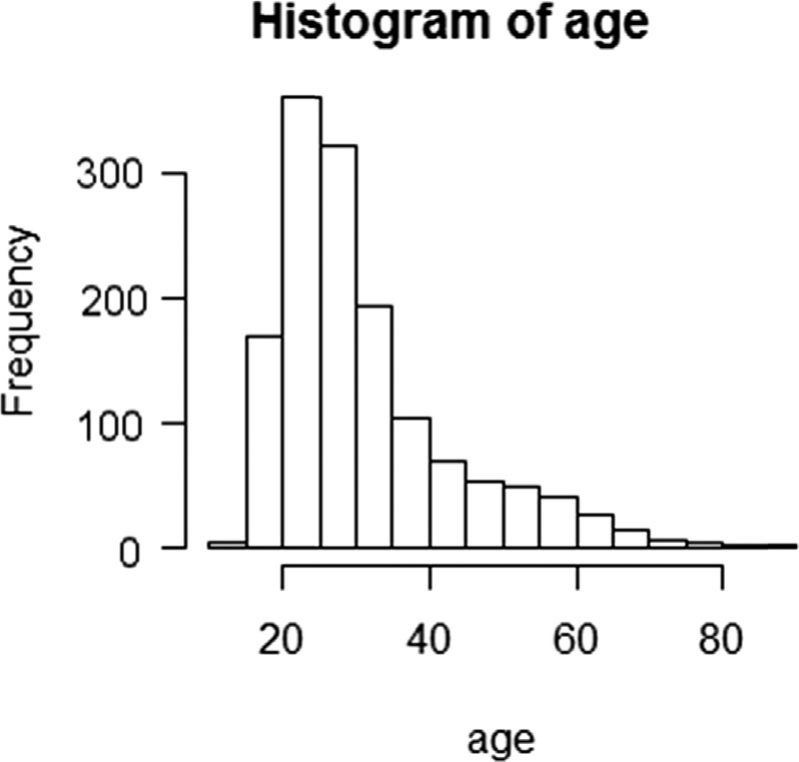
Distribution of participating patients by age.

The age distribution of patients participating in the survey is in Fig. 1.

Discrete (categorical) variables are measured and reported in the survey data set (see Table 1).

## 2 Experimental design, materials and methods

The data can be employed by the multi-category logit models to enable analysis based on baseline-category logits (BCL), for computing probabilities upon events of hypothetical influence. The logic for designing the experiment and thus data set is described as follows. A patient (among *n*) is treated as independent and identical. Each data point has outcome in any of *J* categories for each factor to be investigated. Let $y_{\mathrm{ij}}=1$ if patient *i* has outcome in category *j*, and $y_{\mathrm{ij}}=0$ otherwise. Then, $y_{\mathrm{ij}}=\left(y_{i1},y_{i2},\ldots ,y_{\mathrm{ic}}\right)$ represents a multinomial trial, with $\sum_{j}y_{\mathrm{ij}}=1$. As $n_{j}=\sum_{j}y_{\mathrm{ij}}$ the number of trials having outcome in category *j*, the design is based on the assumption that $\left(n_{1},n_{2},\ldots ,n_{c}\right)$ show a multinomial distribution. Let $\pi_{j}=P\left(Y_{\mathrm{ij}}=1\right)$ denote the probability of outcome in category *j* for each patient, the multinomial probability mass function is

$$p\left(n_{1},n_{2},\ldots ,n_{c}\right)=\left(\frac{n!}{n_{1}!n_{2}!\cdots n_{c}!}\right)\pi_{1}^{n_{1}}\pi_{2}^{n_{2}}\cdots \pi_{c}^{n_{c}},$$

where $\sum_{j}n_{j}=n$. As $\pi_{j}\left(x\right)=P\left(Y=\mathrm{j|}x\right)$ and $\sum_{j}\pi_{j}\left(x\right)=1$, data are grouped into J categories of Y as multinomial with corresponding sets of probabilities $\left\{\pi_{1}\left(x\right),\ldots ,\pi_{j}\left(x\right)\right\}$. Thus, each response is aligned with a baseline category.

$$\ln\frac{\pi_{j}\left(x\right)}{\pi_{J}\left(x\right)}=\alpha_{j}+\beta_{j}^{\prime }x,\;j=1,\ldots ,J-1.$$

BCL models measure the effects of **x** (*J*–1) logits, which in general vary according to the response paired with the baseline category, providing for parameters for these logits.

$$\ln\frac{\pi_{a}\left(x\right)}{\pi_{b}\left(x\right)}=\ln\frac{\pi_{a}\left(x\right)}{\pi_{J}\left(x\right)}-\ln\frac{\pi_{b}\left(x\right)}{\pi_{J}\left(x\right)}$$

The empirical dataset will then be used to evaluate Pearson-type likelihood ratio test statistics ($X^{2},G^{2}$) for goodness-of-fit, following a multivariate generalized linear model (GLM) estimations. Technical details for practically estimating multinomial logistic models is provided in Ref. [2]. Applied analysis can be performed in R (see [3]). Practical uses of survey data can be referred to Ref. [4].

Some possible questions and hypotheses worth testing of, using the data set [1], is in Table 2.

The following short R commands help create the data set provided in the file named “Rq1.1.csv” (see [1]):

**Table:** 

>med=read.csv(“E:/DrVuong/Med/Data/20151230Med.csv”, header=T)
>attach(med)
>x11.12.43=xtabs(~x11.convrel+x12.convexp+x43.info)
>ftable(x11.12.43)

Database in file name “Rq1.1.csv” is displayed in Table 3.

In the same way, a contingency table for the distribution of patients who relied on information from friends/relatives and mass media sources is provided in Table 4a.

One example of the analysis is to compute response probabilities from multinomial logits, i.e., $\left\{\pi_{j}\left(x\right)\right\}$, using $\pi_{j}\left(x\right)=\frac{\exp\left(\alpha_{j}+\beta_{j}^{\prime }x\right)}{1+\sum_{h=1}^{J-1}\exp\left(\alpha_{h}+\beta_{h}^{\prime }x\right)}$; with $\sum_{j}\pi_{j}\left(x\right)=1$; $\alpha_{J}=0$ and $\beta_{J}=0$. In the following example, a short R command (Table 4b) is used for estimating multinomial logistic regression with independent variables are “x11.convrel,” “x12.convexp” and the dependent variable is: “x43.info” with a subset of data named Rq1.1.csv.

The above estimation yields coefficients and associated statistics that are reported in Table 5.

Table 6 shown below reports the full empirical distributions of probabilities over different categorical values of factors "x12.convexp" and "x11.convrel."

As a familiar practice, when facing difficulty in accessing expert counseling, Vietnamese patients choose to consult with family members and close friends. Likewise, the estimated coefficients from multinomial logistic regression with independent variables are "x11.convrel," "x13.convint" and the dependent variable is:

In this example, computed probabilities show the effects of both information from friends/relatives and from mass media/Internet on patients’ data sufficiency. Such empirical probabilities are provided in Table 8, using the relationships established in the estimated coefficients of Table 7.

**Fig. 2:**
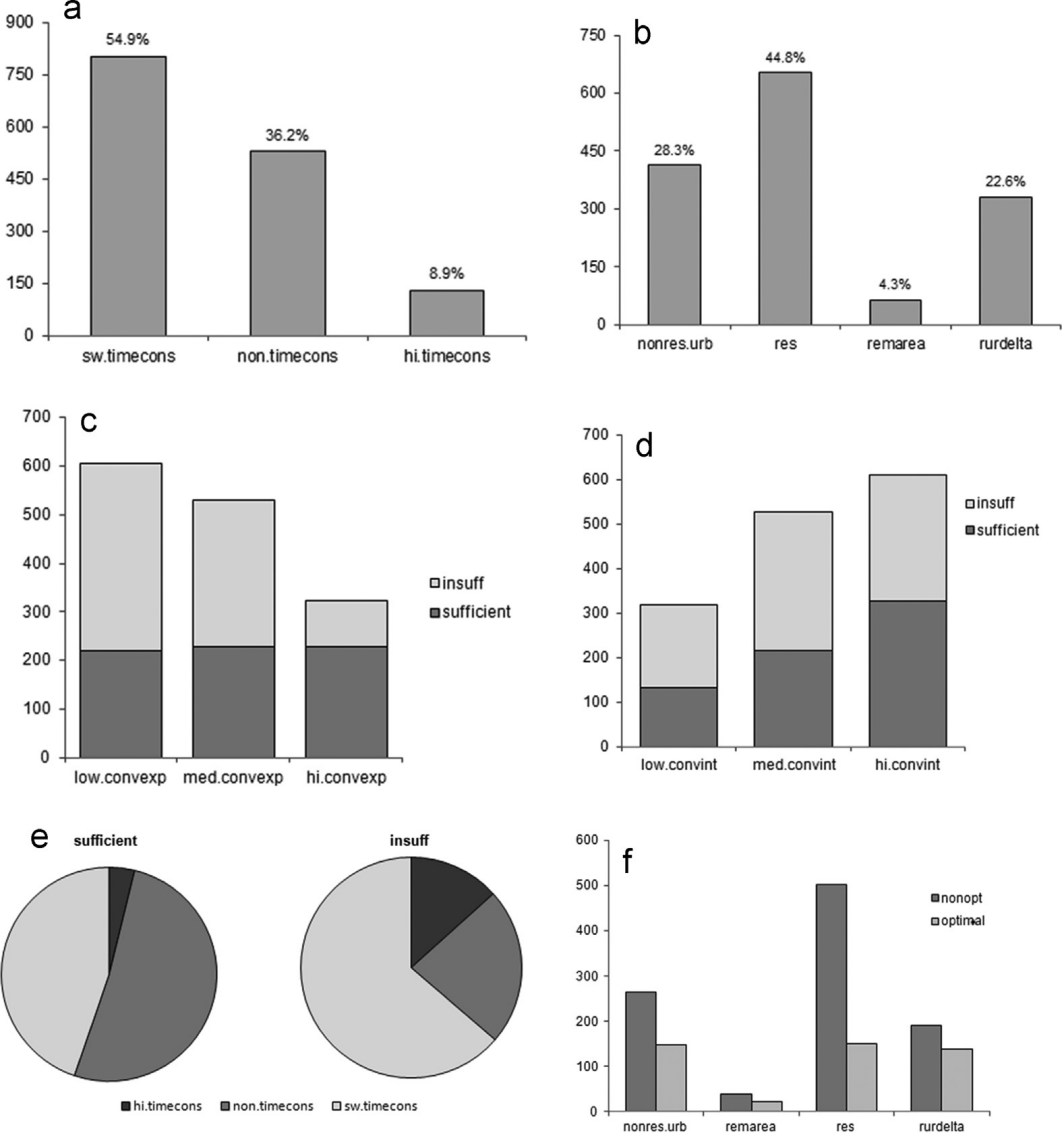
Some graphs from the raw data.

Fig. 2 below is drawn using computed values in Table 7, Table 8 with respect to the changing sociocultural value in the society [5].

**Fig. 3:**
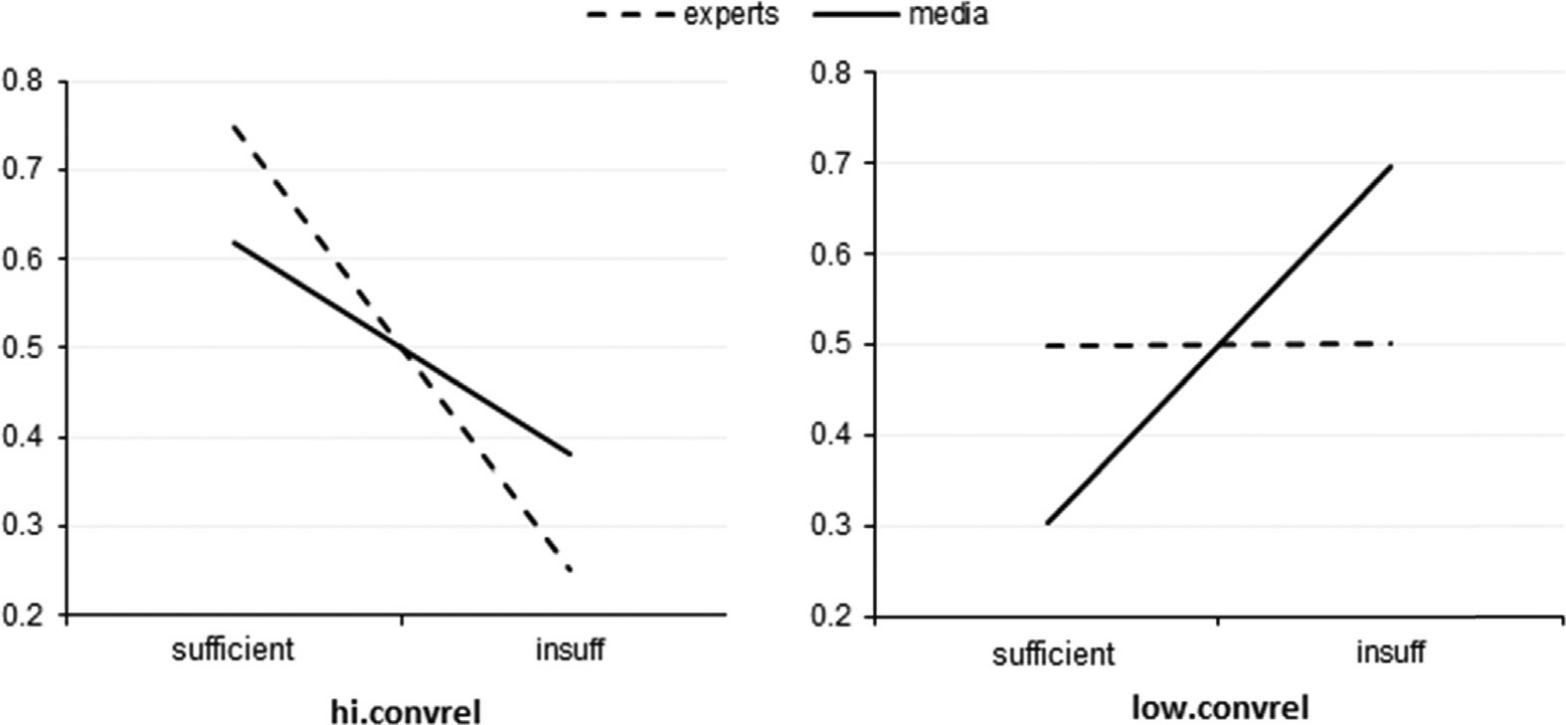
Probabilities of data sufficiency for patients with good access to expert (dash) and to mass media/Internet (solid), with(out) access to friends/relatives.

The changing shapes of the graphs in Fig. 3 show that the positive effect of expert counseling is stronger than that of mass media/Internet, and friends/relatives information source is critically important.

## Figures and Tables

**Table:** 

Health care provider	Obs	Health care provider	Obs
Bach Mai	231	Military 198	15
Viet Duc	108	Hospital E	28
Polyclinic 125 Thai Thinh	61	Military 103	13
Hospitals of Obstetrics and Gynecology	53	Ministry of Construction Hospital	13
Military 108	39	Hospital of Geriatrics	13
Hanoi University of Health Hospital	30	Ministry of Transport Hospital	11
Saint Paul Hospital	28	Ha Dong Polyclinic	11
Thanh Nhan Hospital	27	Hospital of Pediatrics	9
Post Hospital	24	Hospital of Tropical Diseases	6
Institute of Dermatology	18	Others	721

**Table 1:** Categorical variables of the data set.

Coded name	Explanation	Values
Sex	Gender	Male, female
x11.convrel	Information source from friends/relatives	Highly convenient (hi.convrel), somewhat convenient (med.convrel), inconvenient (low.convrel)
x12.convexp	Advice from health care expert counseling	Easy access (hi.convexp), somewhat difficult (med.convexp), difficult (low.convexp)
x13.convint	The Internet source	Easy and convenient (hi.convint), somewhat limited but still available (med.convint), limited and difficult (low.convint)
x21.belfrel	Patients’ trust in information from friends/relatives sources	Believe (bel), only for reference when needed (ref)
x22.belfexp	Patients’ trust in expert information and medical advice	Believe (bel), only for reference when needed (ref)
x23.belfint	Patients’ trust in the Internet information/data source, as well as mass media sources	Believe (bel), only for reference when needed (ref)
x3.ser115	Actual use of the 115 emergency hot-line medical service	Yes, no
x41.time	Representing level of time consumption	Non time-consuming (non.timecons), somewhat time-consuming but acceptable (sw.timecons), and highly time-consuming (hi.timecons)
x42.labor	The labor cost for acquiring information	Low.cost, med.cost, hi.cost
x43.info	The perceived value of information (i.e., subjective assessment of sufficiency) for choosing a health care provider	Information is sufficient for making a good decision (sufficient), information is insufficient for making a good decision (insuff)
x51.cost	Degree of importance of provider’s cost in patient’s choice	Decisive, indecisive
x52.profess	Degree of importance of provider’s professional reputation in patient’s choice	Decisive, indecisive
x53.services	Degree of importance of provider’s services in patient’s choice	Decisive, indecisive
x6.valid	post-treatment assessment of whether a patient’s choice was the best available	Optimal, nonopt
x7.SES	patients’ socio-economic status	Poor, nonpoor
x8.place	The residency status of a patient	Resident (res), non-resident from other urban areas (nonres.urb), from a rural area in the northern rivers delta regions (rurdelta), remote areas, e.g., mountainous regions (remarea)

**Table 2:** Possible research questions arising from the data set.

What are the effects of accessibility to information (through various sources: friends/relatives, mass media – with a focus on the Internet, – and health care experts) on patients’ perception of information sufficiency when having to make a choice regarding a health care provider? How are these sources of information different in terms of their influence on patients’ perception?
What are the measured effects of time and costs spent by patients on *ex ante* probabilities of acquiring sufficient information for decision-making?
What are the effects of socioeconomic status (SES) and residency status on data/information sufficiency for patients’ decision making?
Are the *ex post* probabilities of making an optimal decision conditional upon accessibility to expert information regarding health care and the level of trust in the expertize provided? Is the effect of mass media/Internet use significant?
In what ways do the costliness of information and trust in expertize affect the outcome of a patient’s choice?
Are the use of 115 Emergency Hot-line counseling and the status of residency having significant impacts on patients’ choice outcomes (optimal vs. non-optimal impacts)?

**Table 3:** Patients’ perception regarding information sufficiency following their access to experts and friends/relatives.

“x11.convrel”	“x12.convexp”	“x43.info”	
		“Sufficient”	“Insuff”
“low.convrel”	“low.convexp”	27	99
	“med.convexp”	8	25
	“hi.convexp”	9	6
“med.convrel”	“low.convexp”	67	164
	“med.convexp”	112	169
	“hi.convexp”	58	23
“hi.convrel”	“low.convexp”	125	123
	“med.convexp”	109	108
	“hi.convexp”	162	65

**Table 4a:** Distribution of patients who rely on information from friends/relatives and mass media/Internet sources, with respect to data sufficiency.

“x11.convrel”	“x13.convint”	“x43.info”	
		“Sufficient”	“Insuff”
“low.convrel”	“low.convint”	11	54
	“med.convint”	10	43
	“hi.convint”	23	33
“med.convrel”	“low.convint”	27	66
	“med.convint”	97	192
	“hi.convint”	113	98
“hi.convrel”	“low.convint”	95	66
	“med.convint”	110	76
	“hi.convint”	191	154

**Table 4b:** R commands for BCL estimation.

>info1=read.csv("E:/DrVuong/Med/Data/Rq1.1.csv", header=T)
>attach(info1)
>contrasts(info1$x11.convrel)=contr.treatment(levels(info1$x11.convrel),base=1)
>contrasts(info1$x12.convexp)=contr.treatment(levels(info1$x12.convexp),base=1)
>fit.info1=vglm(cbind(sufficient,insuff)~x11.convrel+x12.convexp,data=info1,family=multinomial)
>summary(fit.info1)

**Table 5:** Estimating impacts of "relatives/friends" and "expert counseling" on information sufficiency.

	Intercept	"x11.convrel"		"x12.convexp"	
		"low.convrel"	"med.convrel"	"low.convexp"	"med.convexp"

	$\beta_{0}$	$\beta_{1}$	$\beta_{2}$	$\beta_{3}$	$\beta_{4}$
logit(sufficient|insuff)	1.092^***^ [8.412]	–1.098^***^ [–5.568]	–0.531^***^ [–4.472]	–1.253^***^ [–8.182]	–1.027^***^ [–6.634]
Signif. codes: 0 ‘^***^’ 0.001 ‘^**^’ 0.01 ‘^*^’ 0.05 ‘.’ 0.1; *z*-value in square brackets; baseline category for: "x11.convrel": "hi.convrel"; and "x12.convexp": "hi.convexp." Residual deviance: 8.79 on 4 d.f.					

**Table 6:** Empirical probabilities computed for RQ1.

"x43.info"	"Sufficient" (a)			"Insuff" (b)		
"x11.convrel"| "x12.convexp"	"low.convexp"	"med.convexp"	"hi.convexp"	"low.convexp"	"med.convexp"	"hi.convexp"
"low.convrel"	0.221	0.263	0.499	0.779	0.737	0.501
"med.convrel"	0.334	0.386	0.637	0.666	0.614	0.363
"hi.convrel"	0.460	0.516	0.749	0.540	0.484	0.251

**Table 7:** Estimating impacts of friends/relatives and mass media/Internet on data sufficiency.

	Intercept	"x11.convrel"		"x13.convint"	
		"low.convrel"	"med.convrel"	"low.convint"	"med.convint"

	$\beta_{0}$	$\beta_{1}$	$\beta_{2}$	$\beta_{3}$	$\beta_{4}$
logit(sufficient|insuff)	0.484^***^ [5.036]	–1.317^***^ [–6.860]	–0.652^***^ [–5.595]	–0.388^**^ [–2.696]	–0.370^**^ [–2.976]
Signif. codes: 0 ‘^***^’ 0.001 ‘^**^’ 0.01 ‘^*^’ 0.05 ‘.’ 0.1 ‘ ’ 1, *z*-value in square brackets; baseline category for: "x11.convrel": "hi.convrel"; and "x13.convint": "hi.convint". Residual deviance: 25.45 on 4 degrees of freedom					

**Table 8:** Empirical probabilities of data sufficiency following access to friends/relatives and mass media/Internet sources.

"x43.info"	"Sufficient"			"Insufficient"		
"x11.convrel"| "x13.convint"	"low.convint"	"med.convint"	"hi.convint"	"low.convint"	"med.convint"	"hi.convint"
"low.convrel"	0.228	0.231	0.303	0.772	0.769	0.697
"med.convrel"	0.364	0.369	0.458	0.636	0.631	0.542
"hi.convrel"	0.524	0.528	0.619	0.476	0.472	0.381

## Appendix A

A1. Breakdown of observations by hospitals.
