# Supplementary material for: Data on Vietnamese patients׳ behavior in using information sources, perceived data sufficiency and (non)optimal choice of health care provider
Source: Data Brief. 2016 May 10;7:1687–95. doi: 10.1016/j.dib.2016.04.066 (PMC5063815; doi:10.1016/j.dib.2016.04.066)
Supplement: Supplementary file 1 — Supplementary material [file mmc1.pdf]

The Statement on Conflict of Interest

I the undersigned and author of the manuscript:

Data on Vietnamese patients' behavior in using information sources, perceived data sufficiency and (non)optimal choice of healthcare provider

hereby state that I have not conflict of interests, now or potentially, with the survey and the dataset and the publishing process of this data article.

I received no funding from any institutions.

The sole purpose of this data survey process is to make a serious research attempt into the topic that the article describes.

I will take all responsibilities for any misstatement or wrong declaration in this.

Quan Hoang Vuong, Ph.D.
Centre Emile Bernheim
Universite Libre de Bruxelles
Brussels B-1050, Belgium
email: qvuong@ulb.ac.be

Quan Hoang Vuong

Digitally signed by Quan Hoang Vuong
DN: cn=Quan Hoang Vuong, o=Universite Libre de Bruxelles, ou=Centre Emile Bernheim, email=qvuong@ulb.ac.be, c=BE
Date: 2016.04.19 14:48:39 +07'00'
